# The oldest psyllipsocid booklice, in Lower Cretaceous amber from Lebanon (Psocodea, Trogiomorpha, Psocathropetae, Psyllipsocidae)

**DOI:** 10.3897/zookeys.130.1430

**Published:** 2011-09-24

**Authors:** Dany Azar, André Nel

**Affiliations:** 1 Lebanese University, Faculty of Sciences II, Department of Natural Sciences, Fanar, Fanar – Matn – P.O. Box 26110217, Lebanon; 2 CNRS UMR 7205, CP 50, Entomologie, Muséum National d’Histoire Naturelle, 45 rue Buffon, F-75005, Paris, France

**Keywords:** New genus, new species, Psyllipsocidae, Lower Cretaceous, Lebanese amber

## Abstract

*Libanopsyllipsocus alexanderasnitsyni* **gen. et sp. n.,** of Psyllipsocidae is described and figured from the Lower Cretaceous amber of Lebanon. The position of the new taxon is discussed and the fossil is compared to other psyllipsocids. The species represents the earliest record of the family Psyllipsocidae.

## Introduction

The Psocodea (Psocoptera + Phthiraptera) is a rather small order with about 10,000 valid extant species. Earlier, fossils from the Permian were assigned to the Psocodea but the attribution of these is uncertain. The first unquestionable records of psocodeans are from the Middle Jurassic of China (Huang et al. 2008). The Psyllipsocidae are a relatively small family of trogiomorphan psocathropetan Psocodea, including about 50 extant species in five genera distributed worldwide. Psyllipsocids live in caves, on rock surfaces, and among dead leaves. To date four fossils have been assigned to Psyllipsocidae: *Psyllipsocus eocenicus* Nel et al., 2005 (lowermost Eocene French Oise amber), ?*Psyllipsocus banksi* Cockerell, 1916 (mid-Cretaceous Burmese amber), a ”*Psyllipsocus* sp. (nymph)“ from Lower Miocene Mexican amber (Mockford 1969), and *Khatangia inclusa* Vishniakova, 1975 (Late Cretaceous Taimyr amber) but the last three attributions to Psyllipsocidae are dubious and require revision.

The Lower Cretaceous Lebanese amber is so far the oldest (Neocomian) with complete macroscopic biological inclusions (Azar 2007). Its formation corresponds to the epoch of the origin and rise of flowering plants, a critical period for the study of the evolution, with extinction of many ancient insect groups and emergence of effectively modern lineages. Up to now nine species of psocids have been described from Lebanese amber (Perrichot et al. 2003; Azar and Nel 2004; Grimaldi and Engel 2006; Azar et al. 2008, 2010).

Herein a new psyllipsocid genus and species is described from the Lower Cretaceous amber of Lebanon, representing the earliest record of this family.

## Material and methods

The amber piece containing the inclusion was cut, shaped, and polished, before preparations between two coverslips and in a Canada balsam medium, as described in Azar et al. (2003). The specimen was examined with a Nikon SZ10 stereomicroscope and a Leitz Laborlux-12 compound microscope, both equipped with camera lucida for line drawings. Photographs were made with an Olympus FE-5000 digital camera.

The works of (Smithers (1972, 1990), Lienhard (1998) and Mockford (1993) are followed herein for the systematics of Psocodea. The wing venation nomenclature and terminology of body structures of Smithers (1972) and Lienhard (1998) were adopted.

## Systematic Paleontology

### Suborder Trogiomorpha Roesler, 1944. Infraorder Psocathropetae Pearman, 1936. Family Psyllipsocidae Enderlein, 1911

#### Libanopsyllipsocus gen. n.

urn:lsid:zoobank.org:act:E646B958-D91F-4C29-917D-502035368507

http://species-id.net/wiki/Libanopsyllipsocus

##### Type species.

*Libanopsyllipsocus alexanderasnitsyni* sp. n.

##### Etymology.

After “Libano” (Lebanese in Latin), and “*Psyllipsocus*”, type genus of the family Psyllipsocidae; gender masculine.

##### Diagnosis.

Hypopharynx with two distinct filaments. Macropterous; forewing without pterostigma; M two-branched; areola postica elongate and free; Cu2 and A ending together forming a nodulus. Hindwing with R1 absent; Rs two-branched; M simple; basal cell present. Distal end of tibia with two inner spines; tarsi three-segmented; distal pretarsi with claw with preapical tooth, and without pulvillus and microtrichia.

#### Libanopsyllipsocus alexanderasnitsyni sp. n.

urn:lsid:zoobank.org:act:F79BD9BC-BB80-44B6-AEA4-99748A240347

http://species-id.net/wiki/Libanopsyllipsocus_alexanderasnitsyni

Figs 1 [Fig F2] [Fig F3] [Fig F4] [Fig F5] [Fig F6] [Fig F7] [Fig F8] [Fig F9] [Fig F10] [Fig F11] 12

##### Holotype.

 Specimen n° 30 (male, coll. Azar); provisionally deposited in the Muséum National d’Histoire Naturelle, Paris, France.

##### Locality and horizon.

Lower Cretaceous, Upper Barremian – Lower Aptian, amber of Hammana-Mdeyrij, Caza, Baabda, Mohafazat Jabal Loubnan, central Lebanon.

##### Etymology.

In honor of our friend Prof. Alexandr P. Rasnitsyn, a world authority in entomology and palaeoentomology.

##### Diagnosis.

As for the genus (*vide supra*).

##### Description.

**Figure 1.**
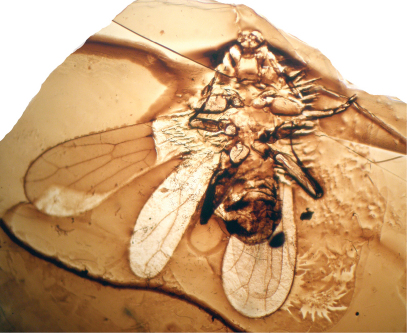
Photograph of habitus of *Libanopsyllipsocus alexanderasnitsyni* gen. et sp. n., holotype, male, specimen number 30.

**Figure 2. F2:**
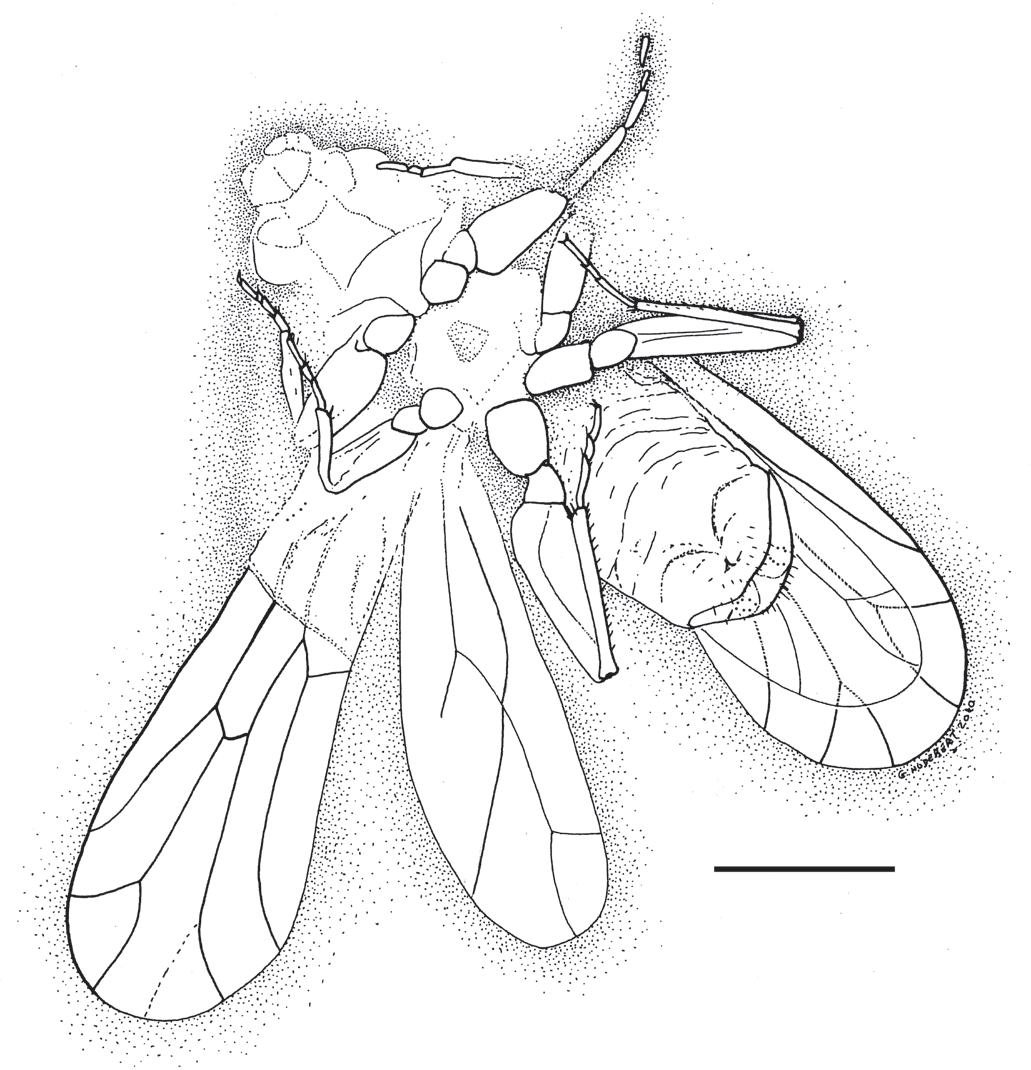
Drawing of habitus of *Libanopsyllipsocus alexanderasnitsyni* gen. et sp. n., holotype, male, scale bar = 0.3 mm.

**Figure 3. F3:**
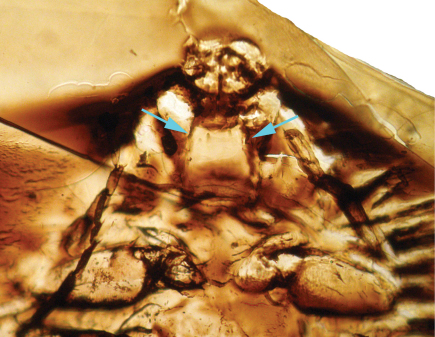
Photograph of hypopharynx filaments (arrows) of *Libanopsyllipsocus alexanderasnitsyni* gen. et sp. n., holotype, male.

**Figure 4. F4:**
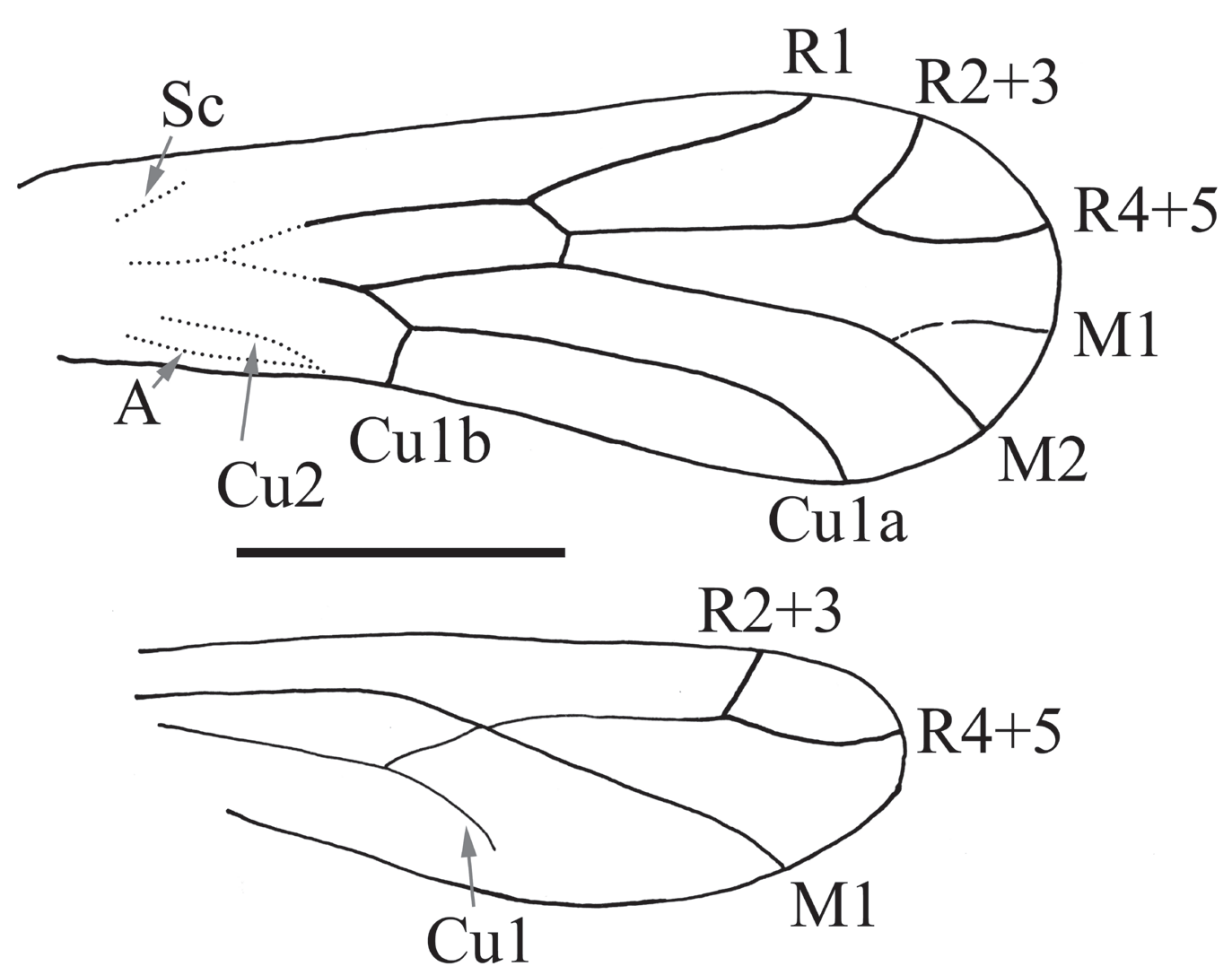
Drawing of wings of *Libanopsyllipsocus alexanderasnitsyni* gen. et sp. n., holotype, male, scale bar = 0.3 mm.

**Figure 5. F5:**
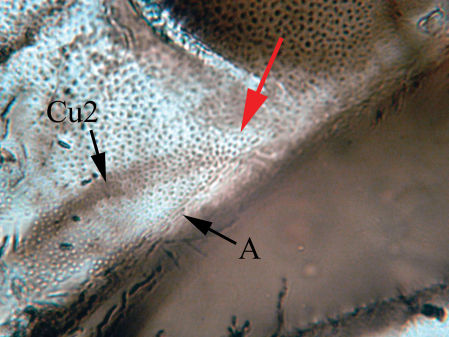
Microphotograph of nodulus, arrow showing the meeting area of Cu2 and A.

**Figure 6. F6:**
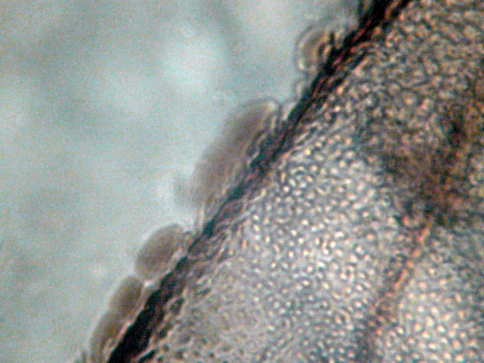
Microphotograph of structure of forewing margin.

**Figure 7. F7:**
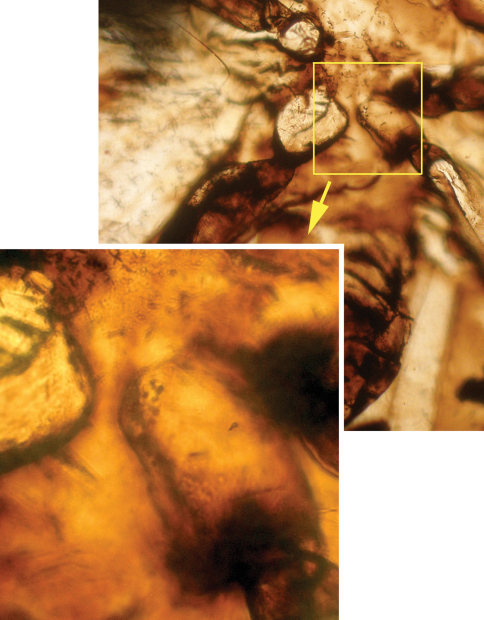
Microphotograph of hind leg coxal rasp (Pearman’s organ).

**Figure 8. F8:**
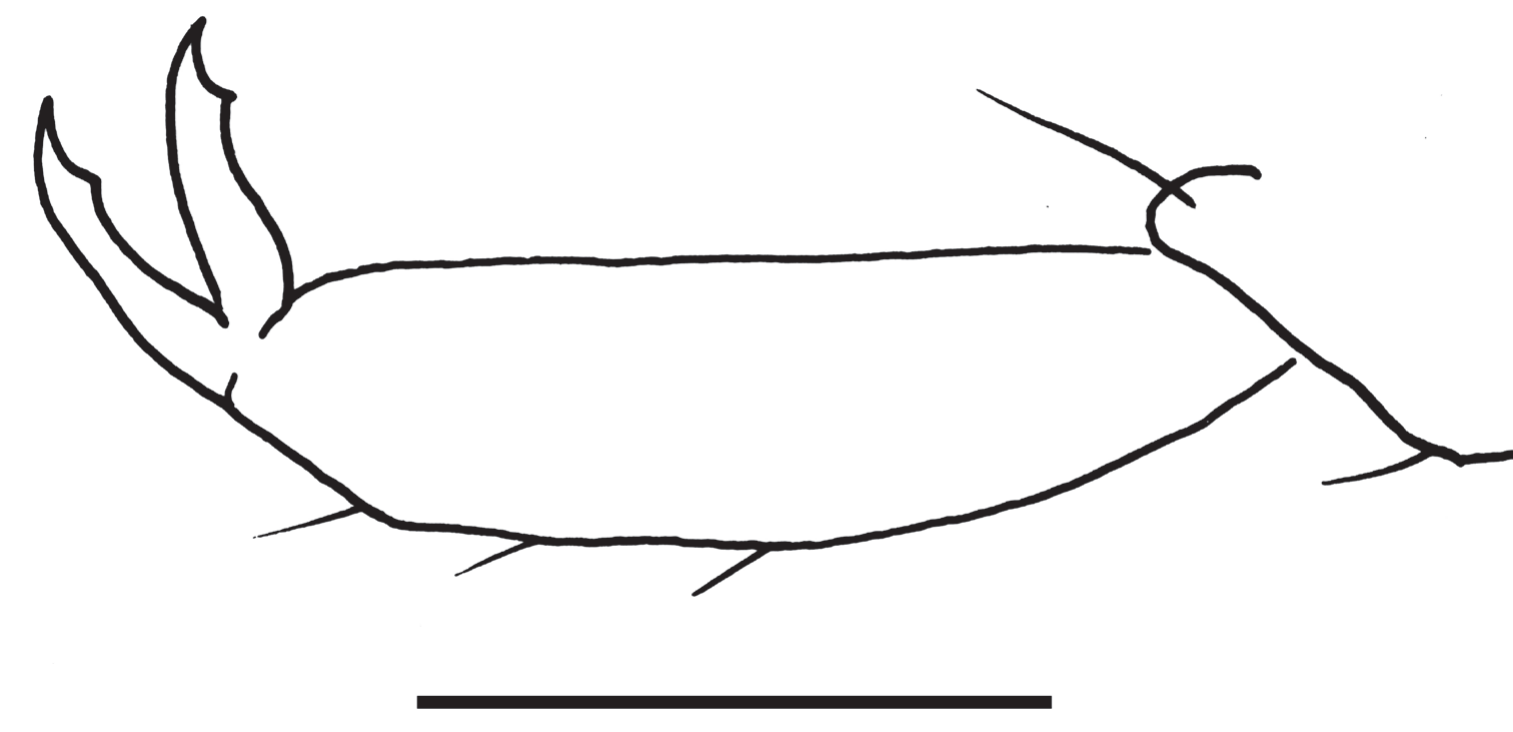
Drawing of pretarsal claw of *Libanopsyllipsocus alexanderasnitsyni* gen. et sp. n., holotype, male, scale bar = 0.03 mm.

**Figure 9. F9:**
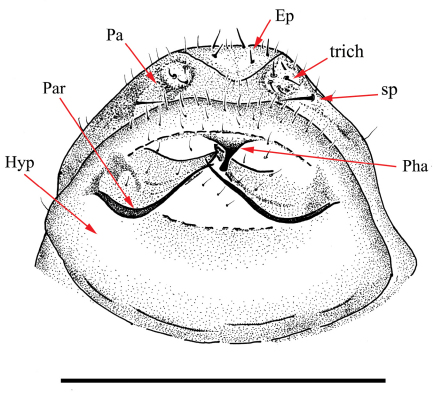
Drawing of aedeagus of *Libanopsyllipsocus alexanderasnitsyni* gen. et sp. n., holotype, male; Ep = epiproct, Hyp = hypandrium, Par = paraproct, par = paramers, Pha = phallosome, sp = anal spine, trich = trichobothria; scale bar = 0.3 mm.

**Figure 10. F10:**
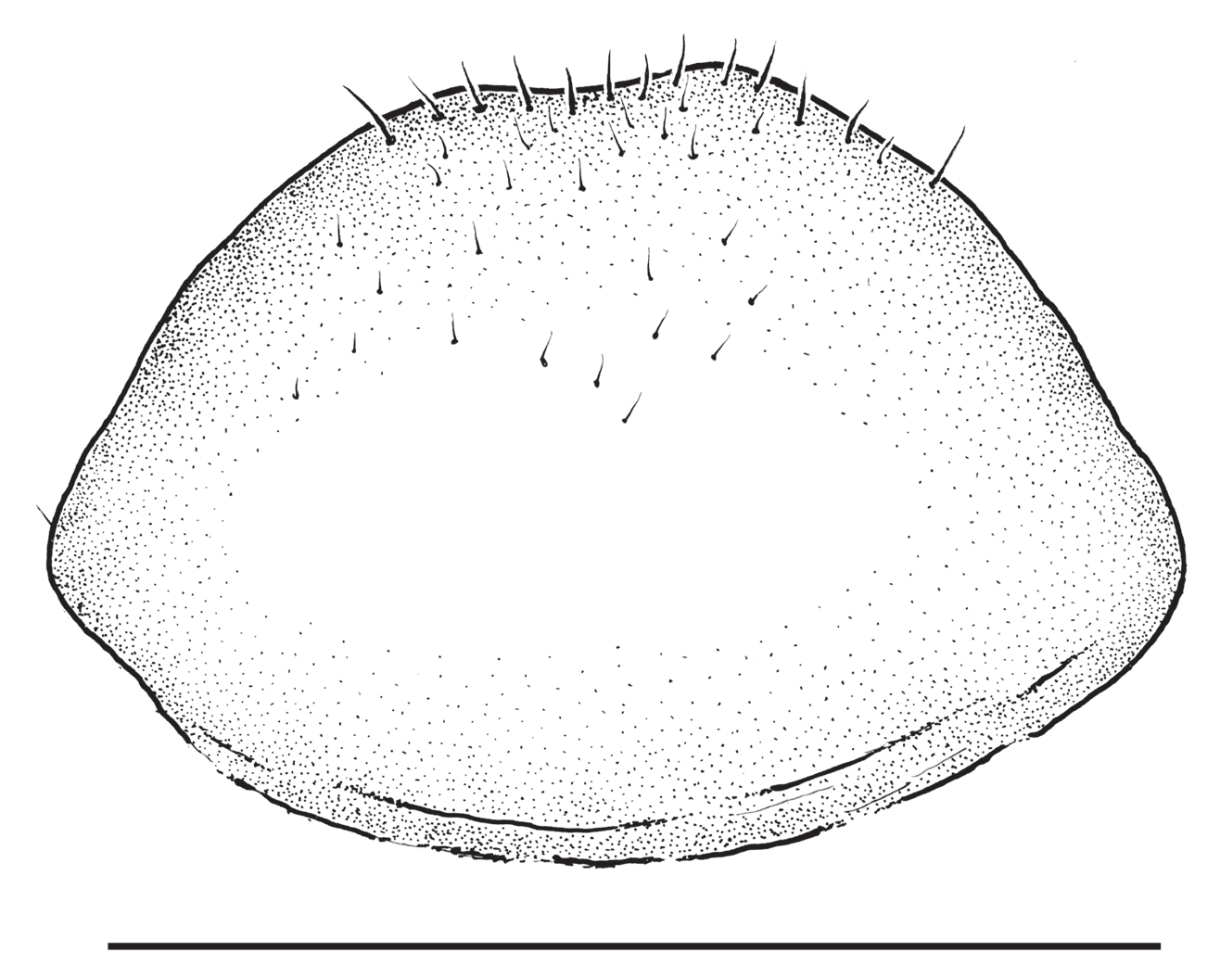
Drawing of hypandrium of *Libanopsyllipsocus alexanderasnitsyni* gen. et sp. n., holotype, male; par = paraproct, ep = epiproct, trich = trichobothria; scale bar = 0.3 mm.

**Figure 11. F11:**
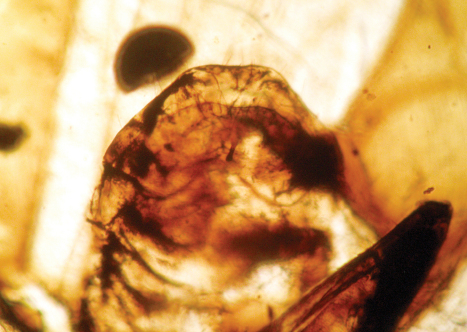
Photograph of hypandrium and aedeagus of *Libanopsyllipsocus alexanderasnitsyni* gen. et sp. n., holotype, male.

**Figure 12.**
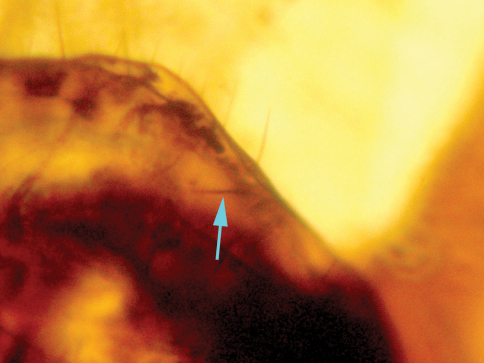
Photograph of paraproct of *Libanopsyllipsocus alexanderasnitsyni* gen. et sp. n., holotype, male; arrow shows the anal spine.

Dorsal parts of head and body not preserved. Body and wings without scales (Figs 1–2). Head elongate (but cannot be measured accurately due to state of preservation), sublingual sclerite and hypopharynx with two distinct filaments (Fig. 3). Forewing transparent (Fig. 4), with infrequent small setae, 0.96 mm long, 0.35 mm broad; apex rounded; Sc badly preserved, not reaching C nor R; R1 reaching costal margin 0.73 mm from wing base; pterostigma absent; Rs separating from R 0.46 mm from wing base; Rs bifurcating into R2+3 and R4+5 at 0.76 mm; R2+3 curved, reaching wing margin 0.83 mm distally; R4+5 curved, reaching wing margin at 0.95 mm distally; a transverse crossvein between Rs and M; fork of M into M1 and M2 0.8 mm from wing base, but one wing is aberrant in having M simple [a frequent teratology in Psyllipsocidae as noted by Lienhard (1998)]; Cu1 bifurcating into Cu1a and Cu1b 0.36 mm from wing base forming a free elongate and narrow areola postica; Cu1a much longer than Cu1b; Cu2 (badly preserved) joining anal vein at posterior wing margin in a nodulus 0.28 mm from wing base (Fig. 5); A strongly convex. Wing margin glabrous (Fig. 6), similar in structure to that illustrated by Lienhard (1998: 111, fig. 31b). Hind wing transparent (Fig. 4), bare and smaller than forewing, about 0.75 mm long, 0.24 mm broad; free or individualized and distinct arm of R1 missing; Rs forked into R2+3 and R4+5 0.16 mm from wing apex; basal cell present; simple M reaching wing margin apically; basal part of wing badly preserved. Hind leg with tibia + tarsi slightly longer than abdomen. Coxal rasp of Pearman’s organ present on hind leg (Fig. 7), but without tympanum (the coxal rasp is preserved on one leg as the coxal area is missing on the other leg). Tibiae with two apical spurs on inner sides; all tarsi three-segmented with first tarsomere longer than others; pretarsal claws with a preapical tooth, without microtrichia nor pulvillus (Fig. 8). Abdomen 0.52 mm long, 0.32 mm broad; telson with epiproct and paraproct as represented in Figs 9–12, paraproct with a rounded hemispherical sensory area of trichobotria, and a spine in its ventral inner side; male genitalia with simple hypandrium, aedeagus with phallosome presenting parameres with curved arms not fused anteriorly as represented in Fig. 9 (it can be seen owing to transparency of sclerites and is comparable in structure to what occurs in other Psyllipsocidae (cf. Lienhard 1998: 112, fig. 32b).

## Discussion

Following the keys of Smithers (1972), Mockford (1993), and Lienhard (1998), *Libanopsyllipsocus alexanderasnitsyni* gen. et sp. n. would fall into Trogiomorpha and can be placed in Psyllipsocidae owing to the following combination of characters, considered diagnostic of the family (Smithers 1972, 1990; Mockford 1993; Lienhard 1998), and present in the current fossil: head elongate; hypopharynx with two distinct sclerotized filaments; presence of nodulus; posterior legs with tibia + tarsi longer than abdomen; tarsi three-segmented; distal pretarsi with claw bearing preapical tooth, without pulvillus; paraproct with anal spine; phallosome with two curved arms not fused anteriorly. Other diagnostic characters of the family Psyllipsocidae could be present but cannot be seen in the specimen owing to the state of preservation, i.e., some parts of the animal are missing.

Our fossil presents a striking and astonishing similarity in some of the wing features (like elongate areola postica and M 2-branched in forewing) with the psocids belonging to the troctomorphan family Pachytroctidae, to the point that any psocidologist at first glance to the specimen would assign it to this family, and as in Pachytroctidae only females could be winged, he would tell directly that this is a female! A minute examination of the specimen shows that the fossil is a male with genitalia structure comparable to those of Psyllipsocidae as figured by Lienhard (1998: 112, fig. 32b) with the very particular conical phallosome directed forwards. Moreover, our fossil could not belong to the Pachytroctidae for several reasons: (1) in the forewing Cu2 and A are meeting together on the wing margin thus forming a nodulus, the absence of this feature being characteristic for Pachytroctidae; (2) presence of Pearman’s coxal organ in hind legs, here too the absence of this feature is characteristic of the Pachytroctidae (Yoshizawa and Lienhard 2010), and even for the whole Nanopsocetae that include this family; (3) presence of distinct separated sclerotized filaments of hypopharynx characteristic of all Trogiomorpha, whereas in Troctomorpha (that enclose Pachytroctidae) these filaments are fused.

Our fossil shows a derived character for its hind wing with M simple, though the majority of the fully-winged extant species of Psyllipsocidae, like most of the trogiomorphan psocids, have hind wings with M forked (a feature considered plesiomorphic in general for the Psocodea).

*Libanopsyllipsocus* gen. n. differs from all modern genera by several features of its wings; i.e., from the genera *Dorypteryx* Aaron, 1899 and *Psocathropos* Ribaga, 1899 by the glabrous anterior margin of the forewing; from *Pseudodorypteryx* García-Aldrete, 1984 and *Dorypteryx* Aaron, 1899 by the broad forewing. *Libanopsyllipsocus* gen. n. resembles mostly the genus *Psyllipsocus* Sélys-Longchamps, 1872 but differs from it in its forewing without a pterostigma and M two-branched, and in its hind wing with R1 absent and M simple. *Libanopsyllipsocus* gen. n. differs from the genus *Khatangia* Vishniakova, 1975 in the absence of a pterostigma and an elongate areola postica in the forewing. All these differences and many others lead us to the proposal of a new genus.

The monophyly of the family Psyllipsocidae is supported by one character of the female genitalia: the spermathecal sac with complex scleritizations at the origin and usually with an accessory vesicle. Molecular analysis also supports the monophyly of the family (Yoshizawa et al. 2006). *Libanopsyllipsocus alexanderasnitsyni* gen. et sp. n. represents the oldest record of the Psyllipsocidae and demonstrates that this family is at least as old as the lowermost Cretaceous, if not older. Unexpectedly some features of this very old fossil are probably derived, viz., the absence of a pterostigma in the forewing and M simple in the hind wing. The discovery of new fossils belonging to this family could bring more information on the polarity and potential homoplasy of some characters in use for the classification, and increase as such our knowledge of the evolutionary history of this lineage.

## Supplementary Material

XML Treatment for Libanopsyllipsocus

XML Treatment for Libanopsyllipsocus alexanderasnitsyni
